# High fascin-1 expression in colorectal cancer identifies patients at high risk for early disease recurrence and associated mortality

**DOI:** 10.1186/s12885-021-07842-4

**Published:** 2021-02-12

**Authors:** Tampakis Athanasios, Tampaki Ekaterini-Christina, Nonni Afrodite, Kostakis D. Ioannis, Posabella Alberto, Kontzoglou Konstantinos, von Flüe Markus, Felekouras Evangelos, Kouraklis Gregory, Nikiteas Nikolaos

**Affiliations:** 1 Clarunis, University Center for Gastrointestinal and Liver Disorders, University Hospital of Basel, Spitalstraße 21, 4031 Basel, Switzerland; 2 Second Department of Propedeutic Surgery, Athens University Medical School, Laiko General Hospital, 17 Agiou Thoma Street, 11527 Athens, Greece; 3 First Department of Pathology, School of Medicine, National University of Athens, Athens, Greece; 4 First Department of Surgery, Athens University Medical School, Laiko General Hospital, 17 Agiou Thoma Street, 11527 Athens, Greece

**Keywords:** Fascin-1, Cytoskeleton, Colorectal cancer, Cancer stem cells

## Abstract

**Background:**

Fascin is the main actin cross-linker protein that regulates adhesion dynamics and stabilizes cell protrusion, such as filopodia. In human cancer, fascin expression correlates with aggressive clinical features. This study aimed to determine the expression patterns of fascin-1 and assessed its prognostic significance in colorectal cancer.

**Methods:**

One hundred eleven specimens of patients with primary resectable colorectal cancer were examined via immunohistochemistry for the expression of fascin-1, and the results were correlated with clinicopathological characteristics and survival data.

**Results:**

Fascin-1 staining displayed strong intensity in the cytoplasm of the colorectal cancer cells and endothelial cells of tumor blood vessels. Moderate to high fascin-1 expression was associated with progressive anatomic disease extent (*p* < 0.001), higher T classification (*p* = 0.007), the presence of lymph node (*p* < 0.001) and distant metastasis (*p* = 0.002), high grade tumors (*p* = 0.002) and vascular invasion (*p* < 0.001). Patients displaying moderate and high fascin-1 expression demonstrated a significantly worse 5-year overall survival [HR; 3.906, (95%CI) = 1.250–12.195] and significantly worse 3-year progression-free survival [HR; 3.448, (95%CI) = 1.401–8.475] independent of other clinicopathological characteristics. Besides, high fascin-1 expression in early-stage cancer only was associated with a dismal prognosis.

**Conclusions:**

High fascin-1 expression in colorectal cancer is an independent negative prognostic factor for survival, increasing the risk for disease recurrence or death almost by sevenfold. Fascin-1 expression could be potentially utilized to identify high-risk patients prone to metastasis already in early-stage disease.

## Background

Cancer stem cells (CSCs) constitute a subpopulation of self-sustaining tumorigenic cells within tumors harboring unique properties such as indefinite potential for self-renewal and maintainance of the tumor [1–6], angiogenic, and immune evasion features [7]. Interestingly, CSCs are considered to be highly resistant to traditional chemotherapy and thus responsible for tumor recurrence [8]. Intriguingly, another hypothesis suggests that metastasis is mediated after cells in a primary epithelial malignancy undergo an epithelial–mesenchymal transition (EMT) [9], which has been associated with CSCs and is also relevant to chemo-resistance [10].

Tumor metastasis represents the major cause of cancer-associated mortality [11], with a total of 53.200 [12] new estimated deaths in 2020 among both sexes with regard to colorectal cancer (CRC). Metastasis, however, is a multi-step process wherein tumor cell migration and invasion are critical steps [13] so that a primary tumor may spread to a secondary tissue [14–16]. Cell migration and invasion proceeds when structural remodeling of the actin cytoskeleton occurs, by forming polymers and bundles to cause dynamic changes in cell shapes [17–19]. Filopodia represent finger-like plasma membrane protrusions whose formation results upon dynamic changes of the actin cytoskeleton [20]. Their role is fundamental regarding the regulation of cell shape and motility events [20].

Fascin is the main actin cross-linker in filopodia [21]. Filopodia formation occurs after fascin binds to 10–30 parallel actin filaments together into straight, compact, and rigid bundles that provide further mechanical stiffness to actin bundles [20, 22]. Previous reports demonstrate that ectopic fascin expression in tumor cells promotes tumor cell migration, invasion, and metastasis [23]. In addition, the upregulation of fascin correlates with the EMT (epithelial to mesenchymal transition) pathway [24].

In this background, the present study aimed to investigate the expression patterns of fascin-1 in CRC and to assess its clinical importance.

## Methods

### Patients and tissues specimens

A total of 111 clinically annotated surgically removed primary CRC samples was retrospectively identified from the database of patients of the 2nd Department of Propedeutic Surgery in Laiko General Hospital in Athens, Greece [25]. The specimens were collected from the established colorectal tumor tissue bank of the Institute of Pathology of the National and Kapodistian University, Athens Medical School, Laiko General Hospital. The Regional Ethics Committee (ethics committee of the National and Kapodistian University, Athens Medical School, Laiko General Hospital) approved the use of tissue samples for translational research purposes. Written informed patient consent was obtained in advance and the study was conducted following the principles of the Helsinki declaration. Results are presented according to the REMARK (Reporting Recommendations for Tumor Marker Prognostic Studies) guidelines [26].

Patients with colorectal cancer were included in the present study and were older than 18 years old, had a documented histopathological report and could give written informed consent for the participation in the study. Patients that underwent a curative surgical resection constituted the study population. Those that manifested with metastatic disease revealed small liver metastases that during surgery were treated with a partial (Wedge/anatomic) liver resection.

### Clinicopathological features

A retrospective analysis followed in a non-stratified and non-matched manner. Annotation included patient age, gender, location, pT/pN classification, tumor grade, histologic subtype, vascular invasion, perineural invasion, the presence of metastasis and the clinical stage of the disease according to UICC (Union for International Cancer Control).

### Immunohistochemistry

Standard procedures were used for the preparation of the formalin-fixed, paraffin-embedded tissue blocks. Staining was done on regular four μm sections after tissue dissection from paraffin embedded tumors. Immunohistochemistry was performed on tissue sections with the standard biotin–avidin technique using a monoclonal antibody against fascin-1. The expression of fascin-1 was analyzed by using the fascin-1 mouse monoclonal IgG_1_ (kappa light chain) antibody (55 K-2:sc-21,743) purchased from Santa Cruz Biotechnology, Heidelberg, Germany. The ImmPRESS REAGENT KIT peroxidase was used for immunohistochemical staining.

Material that was used for the evaluation of the immunohistochemistry was the OLYMPUS BX51 microscope, the OLYMPUS SC30 camera and the UPlan FLN objective lenses. Acquisition of immunohistochemistry images and their transformation to digital data was carried out by using the analysis getIT software provided from OLYMPUS.

### Evaluation of immunohistochemistry

Histopathological evaluation was performed independently by two experienced pathologists in the field (A.N. and E.P.) and by blinding both pathologists to the clinical data of the patients. Immunohistochemistry was applied in a three-step method (avidin- biotin-peroxidase) for determining fascin-1 expression in paraffin tissue-embedded sections of 111 CRC specimens as previously described [27, 28].

**Fig. 1:**
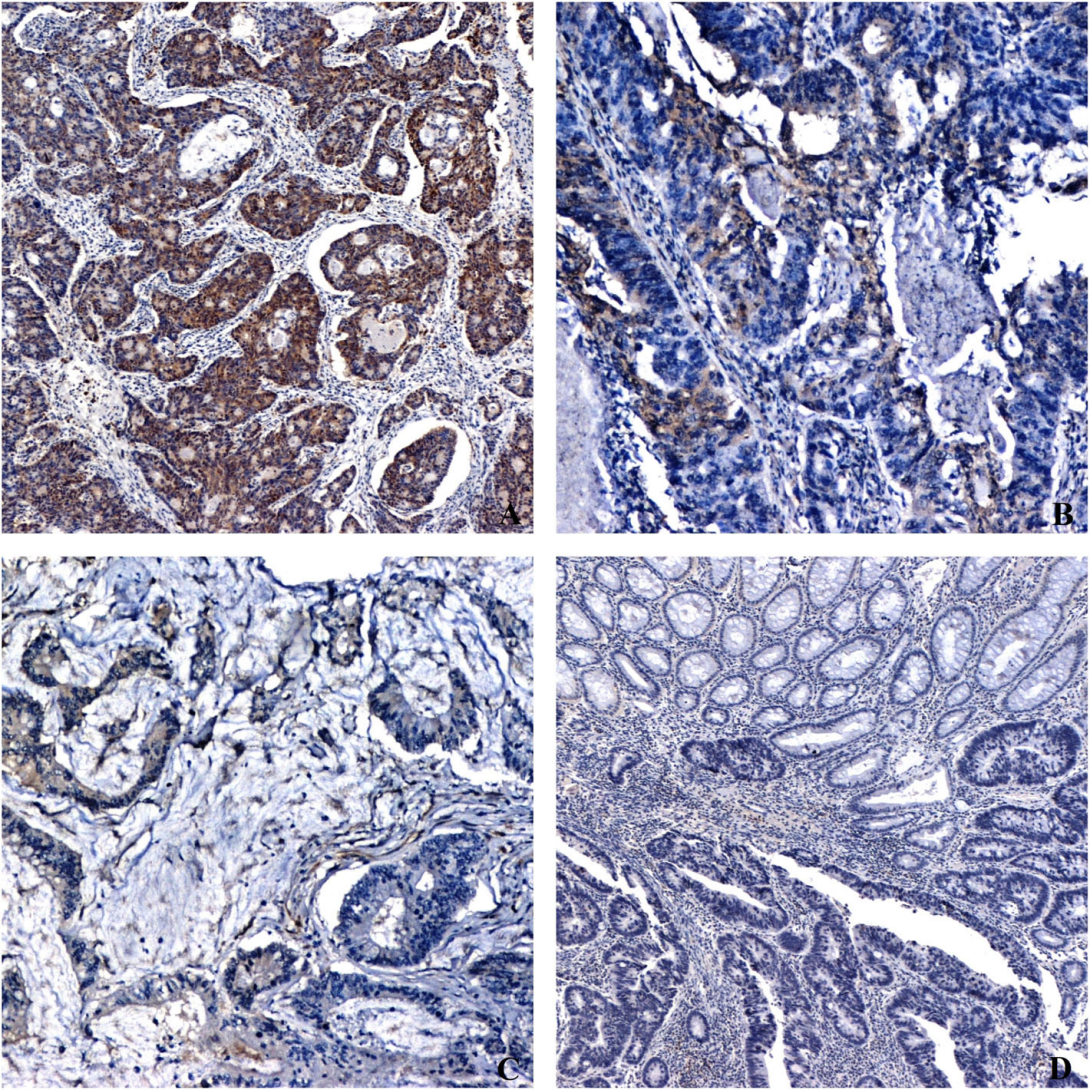
Levels of staining intensity in patients expressing fascin-1 (X100). **a** strong intensity, **b** moderate intensity, **c** weak intensity, **d** negative expression

Expression of fascin-1 was semiquantitatively determined. Staining was evaluated in the cytoplasmic membrane and cytoplasm of tumor cells. The intensity of staining (Fig. 1) was scored as: 0: no staining of the cells, 1: mild staining, 2: intermediate degree of staining, 3: strong staining intensity.

ROC curve (Receiver Operating Characteristics) and X-tile were used to define cut off points and consequently, the levels of expression of fascin-1. Firstly, ROC curve determined 10% of expression of fascin-1 as a significant cut-off point with regard to the clinicopathological characteristics and survival. X-tile software on the other side is a software that has been designed to detect significance when survival data exist. X-tile confirmed the presence of significance with 10% of expression of fascin-1 as a cut-off point for significant survival. However, a second cut-off point (35% of expression of fascin-1) was revealed for the presence of very poor survival. Therefore, two cut-off points were used for the classification of the patients into groups according to the levels of expression of fascin-1.

More specifically, using ROC curve, a specimen exhibiting fascin-1 staining in less than 10% of the cancer cells was defined as a specimen with low expression of fascin-1. Patients displaying fascin-1 expression between 10 and 35% were classified as patients with a moderate expression of fascin-1, whereas patients exhibiting within their specimens fascin-1 expression ≥35% were classified as patients with high fascin-1 expression.

A specimen was evaluated as positive for fascin-1 expression when moderate to high expression of the marker was present and exhibited strong staining intensity.

### Statistical analysis

SPSS IBM statistics software (version 21.0) was used in order to perform the statistical analysis of the present study. The use of *χ2* test assessed correlations between fascin-1 expression and the clinicopathological characteristics. Histogram demonstrated whether stability of the age distribution exists. 2-sided chi-square, Fischer’s exact test and Log-Rank test evaluated the existence of significance. A *p*-value of less than 0.05 demonstrated the presence of statistical significance.

Survival data were graphically demonstrated with Kaplan-Meier curves. Hazard ratios (HR) and 95% confidence intervals (CI) determined the prognostic effects on survival time. Finally, multivariate Cox regression analysis investigated the effect on survival of several clinical parameters related to the fascin-1 expression.

Overall survival (OS) was the length of time in months until the presence of mortality. Time of disease recurrence represented the length of time in months from the operation until the occurrence of the first recurrence manifesting either as local recurrence or distant metastasis. Therefore, progression-free survival (PFS) constituted the time from the operation until the occurrence of disease recurrence or death.

### Outcomes

3-year PFS and 5-year OS were used as the primary outcomes of this study.

## Results

### Baseline descriptive and tumor characteristics of the study population

A total of 111 patients with CRC underwent oncological surgical resection at the second department of Propedeutic Surgery, Laiko General Hospital, National and Kapodistrian University of Athens. The mean age of the study population was 70.9 years (range 42–90 years), and 61.3% were male patients. The primary tumor location was left-sided in 71.2%, with 38.7% representing rectal cancers and right-sided in 28.8% of the patients. The tumor had grown through the muscularis propria and into the subserosa (T3) in 53.2% of the patients. Regarding lymph node metastasis, 53.2% of the patients had no lymph node metastasis, 21.6% presented lymph node metastasis in 1–3 regional lymph nodes, and 25.2% to 4 or more regional lymph nodes. Metastatic disease in terms of resectable liver metastasis was present in 15.3% of the patients. Patients with grade II and III tumors constituted 66.7 and 26.1% of the study population, respectively. Patients with stage I and II disease represented almost half (49.5%) of the study population, whereas patients with stage III and IVA disease were present in 35.1 and 15.3% of the study population, respectively. Vascular, perineural invasion, and mucinous histologic subtype presented in 25.2, 8.1, and 8.1% of the CRC specimens, respectively. Baseline characteristics are summarized in Table 1.

**Table 1:** Descriptive characteristics of the study population

Characteristics	N or mean	(% or range)
**Age, years (median, mean)**	73, 70.9	42–90
**Sex**		
Female (%)	43	38.7
Male (%)	68	61.3
**Anatomic site of the tumor**		
Right-sided (%)	32	28.8
Left-sided (%)	79	71.2
Rectal cancers	43	38.7
**T stage**		
T1 (%)	14	12.6
T2 (%)	23	20.7
T3 (%)	59	53.2
T4 (%)	15	13.5
**N stage**		
N0 (%)	59	53.2
N1 (%)	24	21.6
N2 (%)	28	25.2
**M**		
M0	94	84.7
M1a (liver)	17	15.3
**Tumor grade**		
G1 (%)	8	7.2
G2 (%)	74	66.7
G3 (%)	29	26.1
**UICC**		
Stage IA (%) T1N0	26	23.4
Stage IIA (%) T3N0	26	23.4
Stage IIB-C (%) T4N0	3	2.7
Stage III (%) > N0	39	35.1
Stage IVA (%) > M1	17	15.3
**Vascular invasion (V)**		
No (%)	83	74.8
Yes (%)	28	25.2
**Perineural invasion (Pn)**		
No (%)	102	91.9
Yes (%)	9	8.1
**Mucinous Subtype**		
No (%)	102	91.9
Yes (%)	9	8.1
**Fascin-1 expression**		
< 10%	66	59.5
≥ 10 and < 35%	29	26.1
≥ 35%	16	14.4

### Fascin-1 is strongly expressed in the cytoplasm of the colorectal tumor cells

Fascin-1 expression was observed in the cytoplasm of the cancer cells exhibiting only strong intensity (Fig. 1). Epithelial cells of the adjacent healthy colorectal tissue had no fascin-1 expression. Besides, a moderate to vigorous intensity was observed in the endothelial cells of tumor blood vessels.

According to the cut-off points that were defined, 59.5% of the patients exhibited low expression of fascin-1, 26.1% moderate expression, and 14.4% high expression of fascin-1 (Table 1).

### Correlation of clinicopathological characteristics with the expression of fascin-1

No significant correlations were observed between fascin-1 expression and the presence of perineural invasion (*p* = 0.154) or the presence of mucinous histologic subtype (*p* = 0.481). On the contrary, fascin-1 expression was significantly higher in patients with CRC of clinical stage III and IV compared with stage I and II (*p* < 0.001). Fascin-1 expression was significantly lower in colorectal specimens classified as T1 tumors compared with T2–4 (*p* = 0.007). Lymph node metastasis was significantly associated with patients exhibiting fascin-1 in the CRC specimens (*p* < 0.001). Patients with metastatic disease presented with a significantly higher expression of fascin-1 (*p* = 0.021). Fascin-1 expression correlated with high-grade tumors (*p* = 0.002). Finally, vascular invasion was significantly higher in specimens exhibiting moderate and high expression of fascin-1 (*p* = 0.001). Table 2 summarizes the correlation of the expression of fascin-1 with the clinicopathological characteristics.

**Table 2:** Correlation of fascin-1 expression with clinicopathological characteristics

Colorectal cancer ***N*** = 111	Fascin				***P***
	No or low expression		Moderate to high expression		
	N	% within fascin	N	% within fascin	
**Stage**					
I + II	44	66.7	11	24.4	**< 0.001**
III + IV	22	33.3	34	75.6	
**T**					
1	14	19.8	1	2.2	(T1 vs T2–4) **0.007**
2	13	19.6	10	22.2	
3	37	56.1	22	48.9	
4	3	4.5	12	26.7	
**N**					
0	47	71.2	12	26.7	**< 0.001**
1 + 2	19	28.8	33	73.3	
**M**					
no	62	93.9	32	71.1	**0.002**
yes	4	6.1	13	28.9	
**G**					
1 + 2	56	84.8	26	57.8	**0.002**
3	10	15.2	19	42.2	
**V**					
no	57	86.4	26	57.8	**0.001**
yes	9	13.6	19	42.2	
**Pn**					
no	63	95.5	39	86.7	0.154
yes	3	4.5	6	13.3	
**Mucinous**					
no	62	93.9	40	88.9	0.481
yes	4	6.1	5	11.1	

### Prognostic significance of fascin-1 regarding progression-free and overall survival

**Fig. 2:**
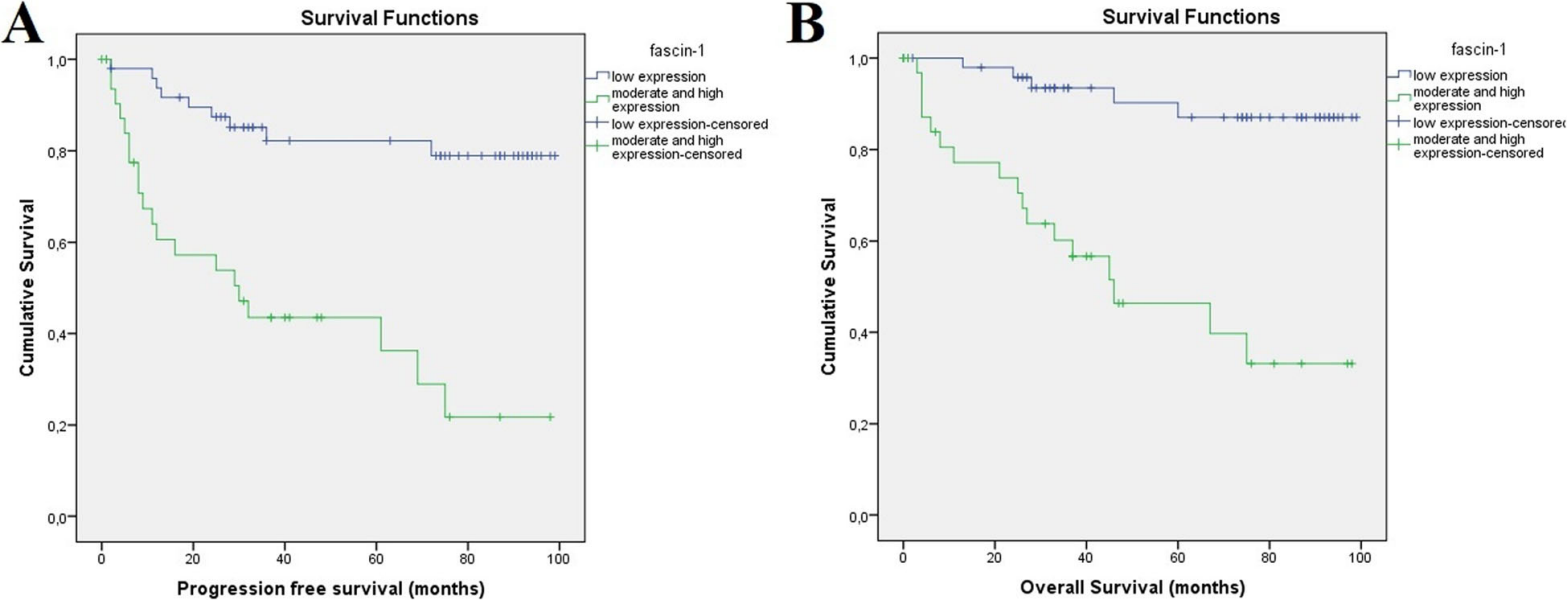
Kaplan Meier curve depicting progression free survival (**a**) and overall survival (**b**) in months for patients with low expression versus moderate and high expression of fascin-1

Median follow up of the patients in the present cohort was 49 months [range 0 (3 patients lost to follow up) to 99 months]. Patients with moderate and high expression of fascin-1 demonstrated significantly higher rates of disease recurrence or death. More specifically, 3-year PFS (Fig. 2) was 43.5%, (95%CI) = 29–57 for patients displaying moderate and high expression of fascin-1 versus 82.2%, (95%CI) = 74–92 for patients with low expression of fascin-1 (*p* < 0.001). Regarding OS, a significantly worse 5-year OS was observed in the group of patients exhibiting moderate and high expression of fascin-1 (Fig. 2) compared to patients with low fascin-1 expression [5-year OS 46.4%, (95%CI) = 39–67, vs 87.1%, (95%CI) = 84–98 *p* < 0.001).

**Fig. 3:**
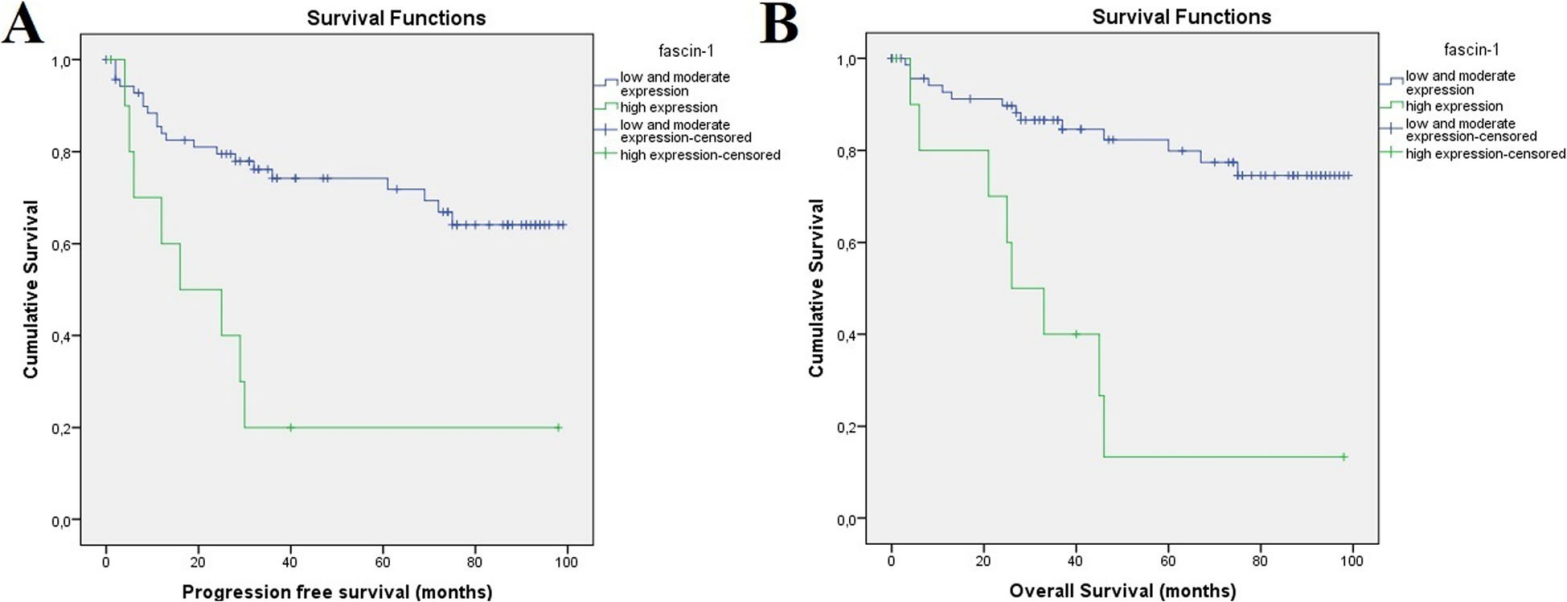
Kaplan Meier curve depicting progression free survival (**a**) and overall survival (**b**) in months for patients with high expression of fascin-1 expression versus patients displaying low and moderate expression of fascin-1

X-Tile software encountered a level of fascin-1 expression of more than 35% (high expression of fascin-1) as a cut-off point for very poor 3-year PFS and 5-year OS. More specifically, 3-year PFS (Fig. 3) reached only 20%, (95%CI) = 11–53 for patients exhibiting high fascin-1 expression versus 75%, (95%CI) = 65–83 for patients displaying low and moderate fascin-1 expression (*p* < 0.001). A poor 5-year OS (Fig. 3) was confirmed for patients with high fascin-1 expression reaching only 13.3%, (95%CI) = 12–53 versus 80%, (95%CI) = 75–90 (*p* < 0.001) for patients with low and moderate fascin-1 expression.

**Fig. 4:**
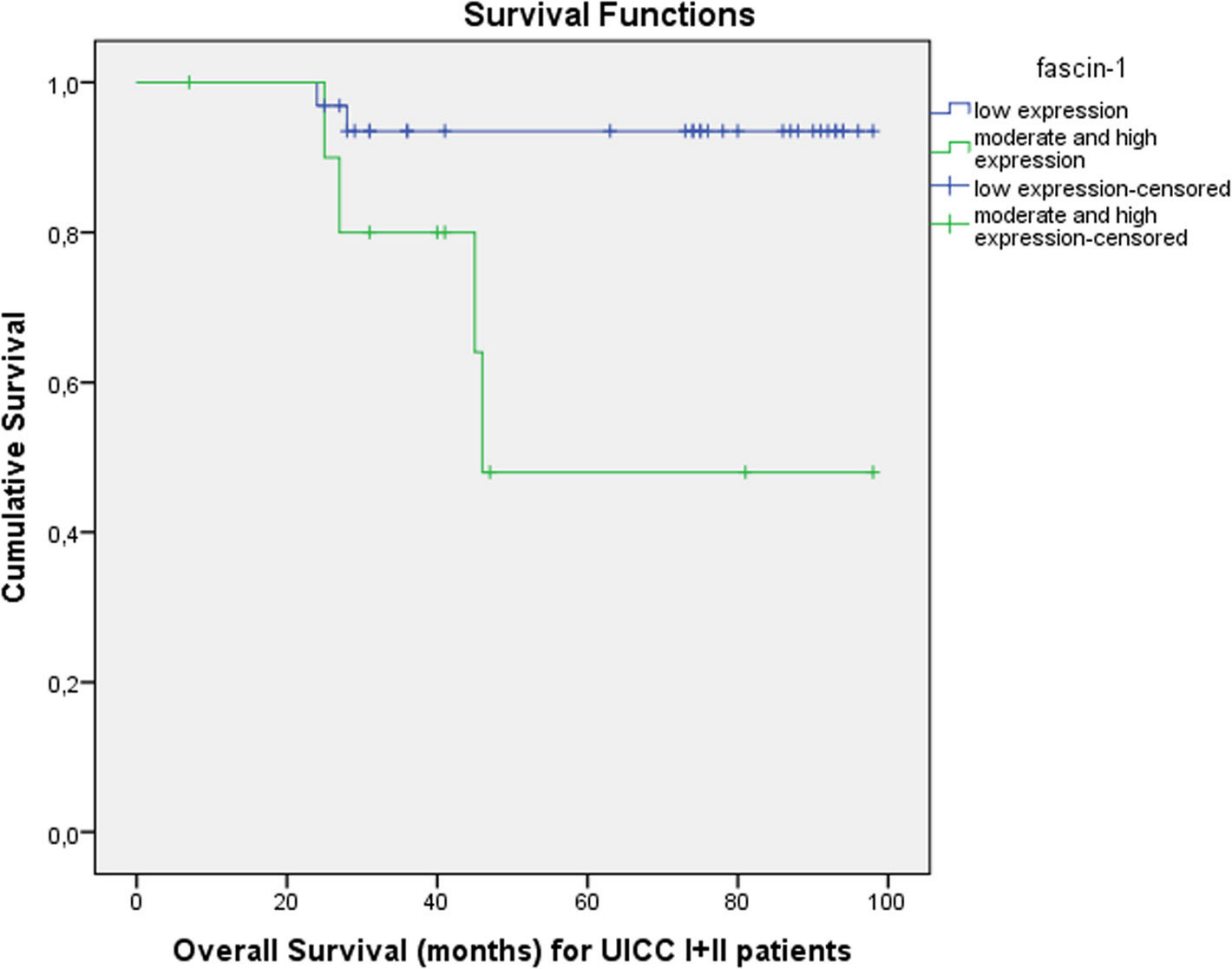
Kaplan Meier curve illustrating overall survival in months for patients with early stage cancer and comparing low versus moderate and high expression of fascin-1

Interestingly, the subgroup analysis comprised only of patients with clinical stage I and II disease demonstrated a significantly worse 5-year OS (Fig. 4) for patients displaying moderate and high fascin-1 expression when compared to patients with a low fascin-1 expression [48%, (95%CI) = 44–68, vs. 93.5%, (95%CI) = 87–99, *p* = 0.007).

Multivariate analysis using the Cox proportional hazards model identified that the risk of occurrence of disease recurrence or death in the first 3 years after operation increases by almost 3.5-fold [HR = 3.448, (95%CI) = 1.401–8.475, Table 3] when patients display a moderate to high fascin-1 expression and by 4-fold in patients with high fascin-1 expression [HR = 4.032, (95%CI) = 1.572–10.309, Table 3] regardless of TNM stage, grade of tumor differentiation, vascular invasion, and perineural invasion. Similarly, the related to fascin-1 expression risk of death in the first 5 years after operation increased by almost 4-fold [HR = 3.906, (95%CI) = 1.250–12.195, Table 4] in patients displaying moderate to high fascin-1 expression and by almost 7-fold [HR = 6.623, (95%CI) = 2.299–19.231, Table 3] in patients with high fascin-1 expression independently of other clinicopathological characteristics.

**Table 3:** Multivariate analysis for progression free survival using cox proportional hazard models

Multivariate Analysis (PFS)	Sig.	Exp(B)	95,0% CI for Exp(B)	
			Lower	Upper
T	**0.010**	0.121	0.024	0.606
N	**0.007**	0.293	0.121	0.714
M	**0.027**	0.334	0.126	0.882
G	0.230	2289	0.593	8839
V	0.550	1391	0.471	4107
Pn	0.559	1454	0.414	5102
Fascin-1 (10%)	**0.007**	0.290	0.118	0.714
Fascin-1 (35%)	**0.004**	0.248	0.097	0.636

**Table 4:** Multivariate analysis for overall survival using cox proportional hazard models

Multivariate analysis (OS)	Sig.	Exp(B)	95,0% CI for Exp(B)	
			Lower	Upper
T	**0.054**	0.218	0.046	1027
N	**0.004**	0.189	0.061	0.586
M	**0.023**	0.276	0.091	0.836
G	0.920	0.933	0.244	3568
V	0.504	0.677	0.216	2126
Pn	0.856	1126	0.312	4066
Fascin-1 (10%)	**0.019**	0.256	0.082	0.800
Fascin-1 (35%)	**< 0.001**	0.151	0.052	0.435

## Discussion

The present study demonstrated that fascin-1 expression correlates with aggressive clinical features, poor 3-year-PFS, and 5-year OS regardless of TNM stage, tumor grade, vascular and perineural invasion. Moreover, patients with early-stage CRC encountered poor 5-year OS when fascin-1 displayed moderate to high expression.

Similar results have been supported previously in the literature. A meta-analysis [29] of solid tumors about fascin expression demonstrated an increased risk of mortality in breast, colorectal, and esophageal cancer. More specifically for CRC patients, two studies [30, 31] in this meta-analysis [29] referring to advanced disease investigated the correlation between fascin expression and time-to disease progression with a total of 354 CRC patients. The pooled random effects hazard ratio was estimated at 2.12 (*p* = 0.05) for mortality, recurrence, or metastasis. However, a moderate to substantial heterogeneity existed among these two cohort studies (*I*^*2*^ = 73%, *p* = 0.06). A strong correlation [29] of fascin expression with lymph node (RR estimate of 1.47, *p* < 0.001 for *N* = 833 patients) and distant metastasis (RR estimate of 1.70, *p* = 0.004 for *N* = 684 patients) was, also, revealed. Interesting to note is the arguable smaller range of heterogeneity (*I*^*2*^ = 6.4%, *p* = 0.38 and *I*^*2*^ = 0%, *p* = 0.83 respectively) among the included studies of the meta-analysis. Chan et al. [32] suggested that there was a stronger correlation with poor overall survival when fascin expression exists in the central region of the tumor rather than the invasive front. Finally, Hashimoto et al. [33] demonstrated the presence of correlation among fascin and Ki-67 in adenomas of the colon, suggesting, therefore, a potential role of fascin in the neoplastic transformation from a low-grade dysplastic lesion to frankly invasive cancer. In our study, fascin-1 expression was significantly higher as the anatomic disease extent progressed.

Although fascin seems to be associated with aggressive clinical features in CRC, the exact mechanisms that mediate fascin dependent cell invasion remain unclear. Studies investigating the role of fascin have established that extracellular signaling pathways regulate its acting-binding properties by acting both through adhesion receptors and receptor tyrosine kinases [34]. Besides, in mouse xenografts, cells from human ovarian cancer cells displaying fascin expression were more tumorigenic, indicating an association of fascin with the proliferative status of the cancer [35]. Jayo and colleagues [36] suggested a role for fascin as a mechano-transducer of cytoplasmic forces that couples F-actin to the nuclear envelope protein nesprin-2. More specifically, this “coupling” regulates nuclear localization and deformation, which contribute to the migration of cells in confined spaces suggesting, therefore, a fascin-mediated structural remodeling of the cytoskeleton. The latter is further enhanced as Jawhari et al. [37] enhance the latter by presenting that fascin positive epithelial cells promote local motility and invasion by altering the organization of actin-based protrusions and focal cell-matrix adhesions.

In the present study, fascin-1 expression was associated with lymph node and distant metastasis and with high-grade tumors. Previous reports demonstrated that high fascin expression correlates with low E-cadherin expression, suggesting, therefore, that as cells progress through the epithelial to mesenchymal transition (EMT), they gain fascin while losing E-cadherin [38, 39]. In addition, the wnt activated LEF-TCF transcriptional signaling pathway constitutes a significant promoter of the EMT pathway and regulates fascin expression [40]. Machesky et al. [24] assumed that when fascin upregulation occurs as part of the EMT pathway, it confers special motility and invasion properties on cancer cells. Furthermore, fascin, via actin stabilization, provides cells powerful invasive properties which enhance their capability to promote metastasis. A stem-cell-like state could potentially explain the aggressive clinical profile of the fascin-1 positive patients. Subsequently, fascin-1 positive cancer cells appear to acquire properties of progenitor cells in colorectal cancer.

Fascin is a stem-cell marker of metastatic carcinomas [41]. A systematic review and meta-analysis consisted of almost 9000 patients (26 studies using immunohistochemistry) with breast, colorectal, gastric, lung and oesophageal cancer demonstrated that high fascin expression is associated with increased mortality and disease progression [29]. More studies revealed that increased expression of fascin leads to a worse clinical course of cancer [34], poor overall survival and poor metastasis-free survival [42]. More specifically for breast cancer, fascin constitutes one of the gene signatures that is associated with breast cancer metastasis to the lung. Despite several actin-cross-linker proteins exist, overexpression of fascin has been found specifically in a series of metastatic tumors [43, 44]. The use of this knowledge has evolved in the era of personalized medicine. It would be desirable to develop small-molecule inhibitors that have the potential to target tumor cell migration. Hunag et al. [41] demonstrated in vitro and in vivo that fascin inhibitors block filopodia formation, tumor cell migration, invasion and metastasis.

Disease recurrence and metastasis are the primary cause of cancer-associated morbidity and mortality. Approximately by 10% of the patients with stage I and 20% of the patients with stage II colon colonic cancer occurs disease recurrence [45]. The effectiveness of adjuvant chemotherapy by balancing toxicity profiles simultaneously with survival for patients with stage II colonic cancer remains controversial as it has been demonstrated to provide minimal survival benefit and is often considered not to be worth of the toxicity of the drugs [46]. Furthermore, previous studies that aimed to identify the subgroup of patients with stage II cancer that are at increased risk of recurrence are not vigorous enough [47] to warrant a clear indication of chemotherapy. In the present study, patients exhibiting high fascin-1 expression displayed a poor overall survival even in early-stage cancer. A stem-cell-like phenotype of fascin-1 positive cancer cells could potentially explain the poor overall survival even in early-stage CRC.

There are limitations to the present study. The retrospective nature of the study requires prospective confirmation with a specific protocol of interventions. In addition, immunohistochemical analysis might underestimate the presence of heterogeneity of fascin expression within the tumor. Nevertheless, our findings do represent a solid basis for the formulation of sound working hypotheses, capitalizing on reliable clinical data.

## Conclusions

In the present study, fascin-1 positive patients displayed aggressive clinical features and demonstrated significantly poor 3-year progression-free survival as well as 5-year overall survival already in early-stage disease. Therefore, fascin-1 could represent a potential target for the development of a tumor profiling test in order to identify patients that are prone to disease recurrence manifested either as local relapse or metastasis. An issue that requires further investigation is whether fascin-1 positive CRC patients with early-stage disease would profit from adjuvant chemotherapy as the current indication is somewhat limited.

## Data Availability

Data are available by the corresponding author upon reasonable request.

## Abbreviations

CI: Confidence intervals
CRC: Colorectal cancer
EMT: Epithelial to mesenchymal transition
HR: Hazard rations
OS: Overall survival
PFS: Progression free survival
REMARK: Reporting Recommendations for Tumor Marker Prognostic Studies
ROC: Receiver Operating Characteristics
UICC: Union for International Cancer Control
