# Correction to: High fascin-1 expression in colorectal cancer identifies patients at high risk for early disease recurrence and associated mortality

**DOI:** 10.1186/s12885-021-07936-z

**Published:** 2021-02-24

**Authors:** Athanasios Tampakis, Ekaterini-Christina Tampaki, Afrodite Nonni, Ioannis D. Kostakis, Alberto Posabella, Konstantinos Kontzoglou, Markus von Flüe, Evangelos Felekouras, Gregory Kouraklis, Nikolaos Nikiteas

**Affiliations:** 1 Clarunis, University Center for Gastrointestinal and Liver Disorders, University Hospital of Basel, Spitalstraße 21, 4031 Basel, Switzerland; 2 Second Department of Propedeutic Surgery, Athens University Medical School, Laiko General Hospital, 17 Agiou Thoma Street, 11527 Athens, Greece; 3 First Department of Pathology, School of Medicine, National University of Athens, Athens, Greece; 4 First Department of Surgery, Athens University Medical School, Laiko General Hospital, 17 Agiou Thoma Street, 11527 Athens, Greece

**Correction to: BMC Cancer 21, 153 (2021)**

**https://doi.org/10.1186/s12885-021-07842-4**

Following publication of the original article [1], the authors identified an error in the author names. The given names and family names were erroneously transposed.

*The correct author names are as follows:*

Athanasios (given name) Tampakis (family name)

Ekaterini-Christina (given name) Tampaki (family name)

Afrodite (given name) Nonni (family name)

Ioannis D. (given name) Kostakis (family name)

Alberto (given name) Posabella (family name)

Konstantinos (given name) Kontzoglou (family name)

Markus (given name) von Flüe (family name)

Evangelos (given name) Felekouras (family name)

Gregory (given name) Kouraklis (family name)

Nikolaos (given name) Nikiteas (family name)

The author group has been updated above and the original article [1] has been corrected.
